# Exposure to Adverse Childhood Experiences Predicts Increased Neurobehavioral Symptom Reporting in Adults with Mild Traumatic Brain Injury

**DOI:** 10.1089/neur.2024.0014

**Published:** 2024-09-20

**Authors:** Dmitry Esterov, Trevor D. Persaud, Jennifer C. Dens Higano, Blake A. Kassmeyer, Ryan J. Lennon

**Affiliations:** ^1^ Department of Physical Medicine and Rehabilitation, Mayo Clinic, Rochester, Minnesota, USA.; ^2^ Brooks Rehabilitation Hospital, Jacksonville, Florida, USA.; ^3^ Mayo Clinic School of Graduate Medical Education, Mayo Clinic College of Medicine and Science, Rochester, Minnesota, USA.; ^4^ Division of Biostatistics, Mayo Clinic, Rochester, Minnesota, USA.

**Keywords:** brain injury, risk factors, adverse childhood experiences, mild TBI, concussion, outcomes

## Abstract

The objective of this study was to understand whether exposure to adverse childhood experiences (ACEs) before 18 years of age predicts increased neurobehavioral symptom reporting in adults presenting for treatment secondary to persistent symptoms after mild traumatic brain injury (mTBI). This cross-sectional study identified 78 individuals with mTBI from 2014 to 2018 presenting for treatment to an outpatient multidisciplinary rehabilitation clinic. Neurobehavioral symptom inventory (NSI-22) scores were collected on admission, and ACEs for each patient were abstracted by medical record review. A linear regression model was used to assess if an individual who experienced at least one ACE before age 18 resulted in significantly different neurobehavioral scores compared with those not reporting any history of an ACE before age 18. Participants who reported at least one ACE before age 18 had significantly increased NSI-22 scores on admission to the rehabilitation clinic compared with patients without history of ACEs (mean difference 10.1, *p* = 0.011), adjusted for age and gender. For individuals presenting for treatment after mTBI, a history of ACEs before age 18 was associated with increased neurobehavioral symptoms.

## Introduction

With over 150,000 resulting emergency room visits each year and sequelae that include both cognitive, affective, and somatic symptoms, mild traumatic brain injury (mTBI) is a significant public health concern.^1^ Although most individuals who sustain an mTBI make a full recovery within days to weeks after injury, a proportion of patients continue to experience persistent symptoms, which is often referred to as post-concussion syndrome (PCS).^2^ A systematic review and meta-analysis found the prevalence of PCS 3–6 months after mTBI to be 18–31.^3^ Moreover, individual longitudinal studies have found that some individuals report ongoing PCS a year after injury,^4^ with others finding that some individuals do not recover fully post mTBI when symptoms persist after three years.^5^

Prior research has identified some risk factors that are associated with prolonged recovery in those who sustain an mTBI,^6,7^ with the goal of identifying individuals at the greatest risk and ideally, preventing the development of chronic symptoms in those individuals.^8^ Studies have shown that pre-injury-related factors may place individuals at an increased risk of prolonged symptoms after mTBI. Several risk factors, including pre-injury life stressful events,^9^ pain, and headache history before injury,^10,11^ as well as pre-injury psychiatric history such as depression,^6^ have been found to be associated with PCS.

To better understand how pre-injury risk factors may be associated with prolonged recovery after mTBI, a pathophysiological model involving the concept of allostatic load has been proposed, wherein cumulative stress before injury predisposes individuals to chronic symptoms after injury.^12,13^ In particular, adverse childhood experiences (ACEs), defined as exposure to abuse and household dysfunction during childhood,^14^ have been recognized as an important influence in the physical, emotional, and cognitive development of individuals, and may be an important risk factor for poor outcome in those who sustain an mTBI.

Studies in the general population have shown that ACEs can impact brain development and function, leading to long-term consequences such as poor mental health outcomes and chronic medical conditions.^15^ The relationship between ACEs and persistent symptoms in mTBI, however, is not well understood. Most studies to date have found a higher incidence of ACEs in those with TBI, though there are limited studies assessing whether ACEs are associated with poor outcome after mTBI.^16^ Understanding the interplay between ACEs and mTBI is important to better understand how these adverse experiences may contribute to negative outcomes in individuals and potentially help better target clinical interventions. The primary aim of this study was to understand whether exposure to ACEs before 18 years of age predicts increased neurobehavioral symptom reporting in adults presenting for treatment secondary to persistent symptoms after mTBI. We hypothesized that in a treatment seeking cohort admitted for multidisciplinary outpatient rehabilitation after mTBI, a history of ACEs before age 18 would be associated with increased neurobehavioral symptom reporting.

## Materials and Methods

### Study setting

This was an observational cross-sectional study. Ethical approval was obtained from the Mayo Clinic Institutional Review Board. Participants gave consent for completion of outcome measures at admission. Medical records for retrospective data collection were reviewed only for individuals who gave authorization for their records to be used for research purposes. Enrollment into this program requires potential participants to be first evaluated by a physician board-certified in Brain Injury Rehabilitation Medicine, and then determined to require ongoing skilled therapy with physical therapy (PT), occupational therapy, and/or speech language pathology, and to complete baseline measures before beginning outpatient therapy.

### Study population

All treatment-seeking patients enrolled into the interdisciplinary brain rehabilitation program from 2014 to 2018 were reviewed for inclusion into this study. Inclusion criteria required individuals to be at least 18 years of age and enrolled into the outpatient program and have completed outcome measures of interest at admission, with a diagnosis of a TBI as made by the Brain Rehabilitation Medicine Physician. Specifically, inclusion criteria required a diagnosis of either a Probable (consistent with mTBI) or a Possible (consistent with concussive) TBI, based on the Mayo Classification System for TBI severity.^17^ Exclusion criteria included a current or prior history of Mayo Classification Definite TBI (consistent with moderate-severe TBI) or a diagnosis of an acquired non-traumatic brain injury.

### Outcome measures

Demographic information was recorded as part of intake data to the rehabilitation program and included gender, race, education, and age at TBI. In addition, employment at the time of admission was assessed. Injury-related factors included either diagnosis of Possible or Probable TBI, days from injury to admission to the rehabilitation program, mechanism of injury, and prior mTBI. In addition, comorbid conditions diagnosed before mTBI were determined by medical record review. These conditions included a pre-existing history of chronic pain, attention-deficit/hyperactivity disorder (ADHD), post-traumatic stress disorder (PTSD), depression, substance use, sleep disorder history, and migraine headaches. Assessment of pre-existing medical conditions from the medical record included review of physician notes on admission as well as a comprehensive medical record review of patient’s history before admission to the rehabilitation program.

#### Neurobehavioral symptom inventory

The main outcome of interest was the Neurobehavioral Symptom Inventory (NSI-22) total score taken on admission to the outpatient rehabilitation program. The NSI-22 is among the more widely used outcome measures used to reliably characterize symptoms after MTBI.^18^ It consists of 22 symptoms commonly reported after concussion, asking the individual to rate each of the symptoms according to how disturbing the symptom is to life activities using a five-point scale (0 = no symptoms, 5 = severe symptoms).^19^ For this study, the total summated score was used as a primary outcome collected upon enrollment to the outpatient rehabilitation program, and subscales with 4 subcategories (vestibular, somatosensory, cognitive and affective) analyzed as well.

#### Adverse childhood experiences

The methodology for identifying ACEs in this study was based on a prior population-based study by Gazzuola Rocca et al.^20^ Each question from the original 10-question ACE survey^14^ was abstracted from the complete electronic medical record of each individual before age 18. This methodology of medical record abstraction of ACEs has been found to have good intra-rater reliability,^20^ as well as good inter-rater reliability in a separate population based study.^21^ Examples of text from the medical record that was abstracted for each ACE question in our study can be seen in the Supplemental Table (Supplementary Table S1), regarding the 10-question ACE items of emotional abuse, physical abuse, sexual abuse, emotional neglect, physical neglect, parental separation, witnessing domestic violence, household substance abuse, household mental illness, and household incarceration.

Medical records were all screened by one investigator (T.P.) who was blinded to outcomes. Inter-rater reliability was assessed by having another investigator (J.D.H.), blinded to the results of the first review, screen 20 participant medical records. Once the data were collected, they was then sent to a third investigator (D.E.), who assessed the agreement between raters using Cohen’s kappa coefficient. The agreement on the presence or absence of each ACE item (yes/no for each of the 10 questions) based on a subset of 20 individuals (200 total data points) was 90% with a kappa statistic of 73% (95% confidence interval [CI], 62–84%) indicating substantial agreement.

#### Data analysis

Continuous variables are summarized as median (1st and 3rd quartile) unless otherwise indicated. Discrete variables are presented as frequency (percentage). Demographic and clinical differences between patients with and without a history of at least one ACE before age 18 were tested using a rank sum test for continuous variables and Pearson’s chi-squared test for nominal variables. A linear regression model was used to assess if an individual who experienced at least one ACE before age 18 results in significantly different NSI-22 admission score or NSI-22 sub-scale scores (vestibular, cognitive, somatosensory, affective), adjusting for sex and age at mTBI. A similar model was used to assess the age- and sex-adjusted association between the number of ACE experiences with those endpoints.

To assess the association of specific ACE items with the admission NSI-22 score, a LASSO model was used with an indicator for each of the 10 ACEs, adjusting for age and gender. The penalty function was only applied to the ACE variables, whereas age and gender coefficients were not penalized. The penalty function helps to highlight effects that might be non-trivial by eliminating some effects that may be noise (resulting in an effect of 0). Mediation analysis was used to assess how the estimated effect of prior ACE is changed by accounting for other patient characteristics (occurring post-ACE but before mTBI). Only mediator variables that were shown to have significant differences between individuals with no prior ACEs before age 18 and in those with ACE before 18 were included in the analysis. Estimates were calculated by taking the difference of prior abuse estimate from the model including age/gender adjustment to a model using prior abuse, age, gender, and the specific mediator variable. All hypotheses’ tests and confidence intervals were two-tailed with a 0.05 significance level. Analyses were conducted using R version 4.2 (R Foundation for Statistical Computing, Vienna, Austria).

## Results

Among 691 individuals reviewed, 78 met inclusion/exclusion criteria. Of those, 51 (65%) individuals experienced at least one ACE before age 18. The most common ACEs found were a history of mental illness in the household (*n* = 26, 33%), verbal/emotional abuse (*n* = 25, 32%), physical abuse (*n* = 21, 27%), and sexual abuse (*n* = 20, 26%) (Table 1). Supplementary Figure S1 provides the distribution of total number of ACEs in the sample. Participants between groups were similar in age, diagnosis (proportion of those with Probable TBI vs. Possible TBI), gender, race, education, mechanism of injury, and employment on admission (Table 2). The median days (Q1, Q3) from injury to admission to the outpatient rehabilitation program was similar across both groups, 143 days (59, 334) days in those with no history of ACEs, and 176 (78, 414) days among those with at least one history of an ACE. There were significant differences found between groups in prior diagnoses of a history of PTSD, depression, substance abuse disorder, and sleep disorder, all increased in those with history of ACE exposure (Table 2). We also assessed whether there were differences among groups in those seeking or receiving Workman’s Compensation for the TBI and were involved in litigation secondary to the TBI, whether the TBI was owing to spousal abuse, or whether the individual’s TBI occurred as a victim in a crime. Though there were slightly greater frequencies among those with history of ACE exposure, the differences were not significant (Table 2).

**Table 1.** Summary of Adverse Childhood Experiences Before Age 18

Adverse childhood experience question	Overall (*N* = 78)
Verbal/emotional abuse	
No	53 (68%)
Yes	25 (32%)
Physical abuse	
No	57 (73%)
Yes	21 (27%)
Sexual abuse	
No	58 (74%)
Yes	20 (26%)
Emotional neglect	
No	64 (82%)
Yes	14 (18%)
Physical neglect	
No	71 (91%)
Yes	7 (9%)
Parental separation	
No	61 (78%)
Yes	17 (22%)
Domestic violence	
No	72 (92%)
Yes	6 (8%)
Household substance abuse	
No	59 (76%)
Yes	19 (24%)
Household mental illness	
No	52 (67%)
Yes	26 (33%)
Household incarceration	
No	77 (99%)
Yes	1 (1%)

**Table 2.** Demographic and Clinical Characteristics of Individuals Admitted for Outpatient Interdisciplinary Rehabilitation with No History of Adverse Childhood Experiences Compared with Individuals with at Least One Adverse Childhood Experience Before Age 18

Characteristic	No history of ACEs (*N* = 27)	At least one ACE before age 18 (*N* = 51)	*p* Value
Age at injury			0.65
Median (Q1, Q3)	47 (30, 55)	43 (33, 53)	
Gender			0.14
Female	17 (63%)	40 (78%)	
Male	10 (37%)	11 (22%)	
Diagnosis			0.96
Possible TBI	15 (56%)	28 (55%)	
Probable TBI	12 (44%)	23 (45%)	
Marital status at time of admission			0.13
Married	16 (59%)	21 (41%)	
Single	11 (41%)	30 (59%)	
Race			0.17
Non-White	1 (3.7%)	0 (0.0%)	
White	26 (96.3%)	51 (100.0%)	
Education (years)			0.99
Median (Q1, Q3)	14 (13, 16)	14.0 (13, 17)	
Employed on admission			0.16
No	13 (48%)	33 (65%)	
Yes	14 (52%)	18 (35%)	
Cause of injury			0.135
Assault	0 (0.0%)	5 (10.0%)	
Fall	8 (29.6%)	12 (24.0%)	
MVA	14 (51.9%)	20 (40.0%)	
Struck by object	1 (3.7%)	9 (18.0%)	
Sports	4 (14.8%)	4 (8.0%)	
Litigation involved			0.95
No	26 (96.3%)	48 (96.0%)	
Yes	1 (3.7%)	2 (4.0%)	
Partner abuse			0.29
No	27 (100.0%)	48 (96.0%)	
Yes	0 (0.0%)	2 (4.0%)	
Crime victim			0.31
No	27 (100.0%)	46 (92.0%)	
Yes	0 (0.0%)	4 (8.0%)	
Workers compensation			0.89
No	23 (85.2%)	42 (84.0%)	
Yes	4 (14.8%)	8 (16.0%)	
Days from injury to admission			0.62
Median (Q1, Q3)	143 (59, 335)	176 (78, 414)	
>90 Days from injury at time of admission			0.74
No	10 (37%)	17 (33%)	
Yes	17 (63%)	34 (66%)	
Prior mTBI			0.13
No	21 (78%)	31 (61%)	
Yes	6 (22%)	20 (39%)	
History of chronic pain^a^			0.098
No	20 (74%)	28 (55%)	
Yes	7 (26%)	23 (45%)	
History of ADHD			0.34
No	24 (89%)	41 (80%)	
Yes	3 (11%)	10 (20%)	
History of PTSD			0.025
No	26 (96%)	39 (76%)	
Yes	1 (4%)	12 (24%)	
History of depression			<0.001
No	12 (44%)	2 (4%)	
Yes	15 (56%)	49 (96%)	
History of substance abuse disorder			0.006
No	24 (89%)	30 (59%)	
Yes	3 (11%)	21 (41%)	
History of sleep disorder			0.006
No	21 (78%)	23 (45%)	
Yes	6 (22%)	28 (55%)	
History of migraine headaches			0.40
No	18 (67%)	29 (57%)	
Yes	9 (33%)	22 (43%)	
Number of comorbidities^b^			<0.001
Median (Q1, Q3)	1 (1, 3)	3 (2, 5)	

^a^ Prior diagnoses were abstracted from the Electronic Medical Record and were diagnosed before MTBI.

^b^ Number of comorbidities include history of migraine headache, depression, ADHD, PTSD, sleep disorder, substance abuse, and chronic pain.

TBI, traumatic brain injury; mTBI, mild traumatic brain injury; ADHD, attention deficit hyperactivity disorder; PTSD, post-traumatic stress disorder.

As shown in Table 3, participants who experienced at least one ACE before age 18 had significantly higher NSI-22 scores on admission (mean difference = 9.7, *p* = 0.013). Within sub-categories (vestibular, somatosensory, cognitive and affective), NSI-22 cognitive and somatosensory scores were significantly higher on admission among those exposed to at least one ACE (Table 3). NSI-22 affective and vestibular sub-scores were associated with an increase in those with a history of at least one ACE but were not statistically significant (*p* = 0.062 and *p* = 0.061, respectively). After adjusting for age and gender, NSI-22 total scores continued to be significantly different in patients exposed to at least one ACE before age 18 (mean difference 10.1, *p* = 0.011, 95% CI: 2.5–17.6), along with cognitive (mean difference 2.5, *p* = 0.009, 95% CI: 0.70–4.38) and somatosensory (mean difference 3.5, *p* = 0.014, 95% CI: 0.78–6.22) scores (Supplementary Table S2). We assessed the effect of experiencing multiple ACEs on NSI-22 admission scores. After adjusting for age and gender, there was a significant increase in NSI-22 score for each additional ACE (mean difference 2.85, *p* = 0.001, 95% CI: 1.28–4.42) (Supplementary Table S3).

**Table 3.** Mean Differences in Neurobehavioral Symptom Inventory (NSI-22) Scores on Admission (Total Score and Sub-Categories) Between Those Who Experienced At Least One Adverse Childhood Experience Before Age 18, Compared with No History of Adverse Childhood Experiences Before Age 18

	No history of ACEs (*N* = 27)	At least one ACE before age 18 (*N* = 51)	*p* Value
NSI-22 Total score admission			
Mean (SD)	28.2 (17.6)	37.9 (14.9)	0.013
NSI-22 Cognitive			
Mean (SD)	6.7 (4.3)	9.2 (3.6)	0.009
NSI-22 Vestibular			
Mean (SD)	3.1 (2.8)	4.4 (2.8)	0.061
NSI-22 Somatosensory			
Mean (SD)	6.9 (6.0)	10.2 (5.5)	0.015
NSI Affective			
Mean (SD)	11.6 (7.1)	14.4 (5.7)	0.062

Table 4 presents a LASSO model describing the conditional effect on NSI-22 scores for each ACE item. For example, after accounting for age, sex, and other ACE components, individuals who experienced mental illness in the household are estimated to score 5.8 points higher on the NSI-22 admission score compared with individuals who did not experience mental illness in the household. Participants who experienced domestic violence (mean difference, 6.4), household mental illness (mean difference, 5.8), along with physical neglect (mean difference, 4.0) showed the greatest difference on NSI-22 admission scores after applying a LASSO penalty adjusted for age and gender. History of household incarceration showed a decrease in NSI-22 admission score (mean difference, 6.60); however, only 1 participant reported household incarceration.

**Table 4.** LASSO Model for NSI-22 Admission, Including Each of the 10 Adverse Childhood Experience Items, Adjusting for Age and Gender

Term	Estimate
Intercept	12.10
Age at injury (5 years)	−0.30
Male	1.63
Verbal/emotional abuse	0.59
Physical abuse	2.42
Sexual abuse	0.00
Emotional neglect	2.32
Physical neglect	3.95
Parental separation	3.45
Domestic violence	6.35
Household substance abuse	0.00
Household mental illness	5.78
Household incarceration	−6.60

We performed a mediation analysis including the variables that were significantly different between individuals with at least one ACE before age 18 and those without any history of ACEs. The variables assessed were a history of PTSD, history of mood disorder, history of substance abuse, and history of a sleep disorder. None of these variables significantly mediated the effect of ACE on NSI-22 total score (Supplementary Table S4).

## Discussion

This cross-sectional study of adults presenting for treatment to an outpatient brain rehabilitation clinic with ongoing symptoms after mTBI found that individuals who experienced at least one ACE before age 18 had significantly increased neurobehavioral symptoms on admission when compared with those without a history of an ACE before age 18, when controlled for age and gender. Potential mediators including a pre-injury history of depression, PTSD, substance abuse, and sleep disorder did not significantly change the overall association of NSI-22 total scores on admission. In addition, each additional ACE was significantly associated with a higher NSI-22 score on admission.

Prior studies directly correlating a history of ACEs with neurobehavioral symptoms after mTBI is limited. The majority of research studying this association has found an increased incidence of TBI in those exposed to ACEs,^22–24^ though the impact of ACEs on recovery after TBI is less clear.^16^ One cross-sectional study assessed the association of depressive symptoms and childhood adversity in 75 individuals after mild-moderate TBI. In this sample, regression models showed that higher scores on childhood adversity significantly accounted for variability in depression scores after TBI.^25^ In another cross-sectional study of women residing in a shelter who had experienced at least one episode of domestic abuse, Saadi et al. found that women reporting increased childhood trauma severity reported significantly increased levels of neurobehavioral symptoms based on the Rivermead Concussion Questionnaire; however, there were no significant differences in neurobehavioral symptom severity between women who self-reported a history of TBI and those who did not.^26^ Neurobehavioral symptoms were mediated by PTSD, and the authors concluded childhood trauma may increase the risk of neurobehavioral symptoms at baseline owing to increased risk of depression, anxiety, or PTSD. Our study also found that individuals with a history of ACEs before age 18 had a higher proportion of diagnoses of PTSD, depression, and substance use disorder before injury. However, mediation analysis in our study did not show that these factors significantly mediated the association, suggesting that a history of ACEs was associated with increased neurobehavioral symptom reporting independent of prior diagnoses of depression and PTSD.

Early life stress including ACEs has been found to impact both cognitive functioning and psychological health in the general population,^27,28^ and mechanisms have been proposed to understand this association in those with persistent symptoms after mTBI. Several researchers have proposed a model involving the concept of allostatic load, such that cumulative stress before TBI predisposes individuals to worse outcome after injury through corticolimbic, hypothalamic-pituitary-adrenal, and neuroendocrine dysfunction.^12,29,30^ Exposure to stress particularly in childhood and adolescence has been found to change neuroendocrine hormone levels and decrease immune responses cellularly through glucocorticoid and adrenergic pathways.^13,31^ As it relates to TBI more specifically, studies using animal models have shown dysregulation of the neuro-endocrine response after TBI^32^ and have found that the combination of early life stress and TBI in adulthood resulted in greater abnormalities in corticosterone responses as well as resulting in impaired cognition.^33^ In this context, mTBI may represent a substantial additional allostatic load that may be associated with PCS for individuals with a history of exposure to stress before injury and especially at an early age, though further research is needed to understand this association.^12,13^

There are important potential clinical implications between the association with ACEs and persistent symptoms after mTBI. By better understanding how ACEs are associated with PCS, this may allow for identification of individuals at the greatest risk and ultimately prevent the development of chronic symptoms in those individuals.^8^ However, the ACEs framework was developed for epidemiological research for population-level or structural policies for prevention and is currently recommended at the population level only,^14^ and not as a tool for individual-level intervention.^34^ Although some researchers have called for a “trauma-informed neurology” approach that can more effectively target stress-response physiology through pharmacological treatments and resiliency interventions,^35^ other researchers have cautioned that ACE questionnaires used for individual evaluation could disrupt the clinician/patient relationship and can be stigmatizing such that an individual could be labeled as inherently at risk for poor outcome.^36,37^ Further work is needed to not only better elucidate the association of ACEs and outcome after mTBI but also to better understand the risks/benefits of screening for ACEs in the rehabilitation setting for a given individual.^34,37^

There are several limitations of this study. Our study relied on a retrospective review of reports in the medical record, and thus the frequency of ACEs were likely underestimated, as noted in other studies.^14,20,38^ On the other hand, prior studies have relied on self-report of TBI and ACEs which is prone to recall bias,^26,39^ and retrospective reports of ACEs may provide a more valid account with less false positive reports.^38^ ACEs were inconsistently reported with a precise age of occurrence but rather often reported as broadly occurring in childhood or adolescence, limiting the understanding of whether there is a particular vulnerability period of ACE exposure as have been previously shown.^40^ In addition to limitations of the frequency of ACEs reporting, there are also limitations to frequency of review of the medical record review of co-morbid conditions, where we may have not captured all comorbidities as the participants did not exclusively receive all their care in one healthcare system. This could result in an underestimation of co-morbid conditions, though this estimation remained similar between groups with a history of at least one ACE compared with no history of ACEs.

There are also limitations with the NSI-22 as an outcome measure. Though the NSI-22 is a widely used outcome measure to reliably assess symptom severity after mTBI,^41^ its validity has been questioned.^42^ In particular, though NSI-22 scores are higher in individuals with TBI when compared with controls, studies have shown that NSI-22 scores are also strongly influenced by emotional distress including comorbid psychiatric conditions such as PTSD or depression, regardless of TBI status.^43^ Though our study did not show that these factors significantly mediated the association, this limitation of the NSI-22 should still be acknowledged given prior studies finding that NSI-22 have strong correlations with psychiatric comorbidity. As our sample was a civilian cohort presenting for treatment to a multidisciplinary clinic, our study did not use any additional validity measures. To determine potential bias in over-reporting, several studies in military populations have developed NSI total score cut-points, including Vanderploeg et al., who presented a cut-point of >58.^44^ Among our sample of 78 individuals, 8/78 (10%) reported an NSI-22 score of over 58.

Our sample was a relatively small cohort of individuals seeking treatment in a multidisciplinary clinic for persistent difficulties after mild TBI, rather than a population-based cohort of all individuals with TBI. This cohort included a high percentage of female participants (57/78 – 73%), and thus may not be representative of the general population with TBI. Lastly, this study only assessed outcomes at admission to a rehabilitation program and did not study the effect of treatment for mTBI in a rehabilitation setting. Future studies should better understand the association with history of ACEs and impact of treatment response after mTBI.

## Supplementary Material

Supplementary Figure S1

Supplementary Table S1

Supplementary Table S2

Supplementary Table S3

Supplementary Table S4

## Data Availability

The participants of this study did not give written consent for their data to be shared publicly, so owing to the sensitive nature of the research, supporting data are not available.

## Abbreviations Used

ACE: adverse childhood experience
ADHD: attention-deficit/hyperactivity disorder
mTBI: mild traumatic brain injury
NSI-22: neurobehavioral symptom inventory 22
PCS: post-concussion syndrome
PT: physical therapy
PTSD: post-traumatic stress disorder
TBI: traumatic brain injury
