# Supplementary material for: Exposure to Adverse Childhood Experiences Predicts Increased Neurobehavioral Symptom Reporting in Adults with Mild Traumatic Brain Injury
Source: Neurotrauma Rep. 2024 Sep 20;5(1):874–82. doi: 10.1089/neur.2024.0014 (PMC11462419; doi:10.1089/neur.2024.0014)
Supplement: Supplementary Figure S1 [file neur.2024.0014_Supplementary_Data.pdf]

Supplemental Table 1: Examples from medical record of each individual Adverse Childhood Event item.

Item	Examples of data from the medical record (mix of direct quotation and paraphrasing)
Q1: Verbal/Emotional Abuse: Did a parent or another adult in the household *often or very often*…swear at you, insult you, put you down, or humiliate you? *Or* act in a way that made you afraid that you might be physically hurt?	• Patient describes a very significant history of emotional abuse by his father growing up beginning at age 5 and went on for many years<br>• The patient was verbally abused by one of her step fathers when she was age 13 or 14. Her mother was also abused.
Q2: Physical Abuse: Did a parent or another adult in the household *often or very often*…push, grab, slap, or throw something at you? *Or* ever hit you so hard that you had marks or were injured?	• Patient notes that he was drugged by close family friends that would come to visit his parents and then physically abused.
Q3: Sexual Abuse: Did an adult or person at least five years older than you *ever*…touch or fondle you or have you touch their body in a sexual way? *Or* attempt to touch you or touch you inappropriately or sexually abuse you?	• Record notes, "while with her mother she suffered sexual abuse from age 5-12 then started to live with her father and step-mother whom she said they have had a good relationship with.
Q4: Emotional Neglect: Did you *often or very often* feel that…no one in your family loved you or thought you were important or special? *Or* feel that your family members didn't look out for one another, feel close to one another, or support one another?	• Record notes, "this leads to a discussion about her long history of neglect. The patient's mother was unreliable when she was growing up, and the patient experienced some degree of role reversal."<br>• Patient describes a negative relationship with her mother involving emotional neglect
Q5: Physical Neglect: Did you *often or very often* feel that…you didn't have enough to eat, had to wear dirty clothes, and had no one to protect you? *Or* that your parents were too drunk or high to take care of you or take you to the doctor if you needed it?	• Patient notes that she stopped relying on her mother many years ago, recalling that her mother would forget to pick her up from school with some regularity"
Q6: Parental Separation: Was a biological parent *ever* lost to you through divorce, abandonment, or another reason?	• Patient states parents had divorced long before she could remember<br>• Record states, "the patient's mother and father are not married. The patient's mother has been married and divorced 3 times.

Q7: Witness Domestic Violence: Was your mother or stepmother *often or very often* pushed, grabbed, slapped, or have something thrown at her? *Or was she sometimes, often, or very often* kicked, bitten, hit with a fist, or hit with something hard? *Or ever* repeatedly hit over the course of at least a few minutes or threatened with a gun or knife?	• Record states, "her adoptive mother was loving, supportive, but conservative. Her father was an alcoholic and she recalls her mother being beaten by him."
Q8: Household Substance Abuse: Did you live with anyone who was a problem drinker or alcoholic, or who used street drugs?	• Family history describes alcohol abuse in mother<br>• Record states, "The patient's biological mother has a history of drug addiction."
Q9: Household Mental Illness: Was a household member depressed or mentally ill, or did a household member attempt suicide?	• Obtained from "family history" section of medical record.<br>• Obtained from self report: "the patient reported his maternal grandfather shot himself to death, a maternal uncle and an aunt committed suicide as well."
Q10: Household Incarceration: Did a household member go to prison?	• Record states, "Patient did not have much of a relationship with her biological father, as he spent a lot of time in prison, and has been out of her life."

Supplemental Figure 1: Scatterplot of distribution of ACEs in the sample (N = 78)

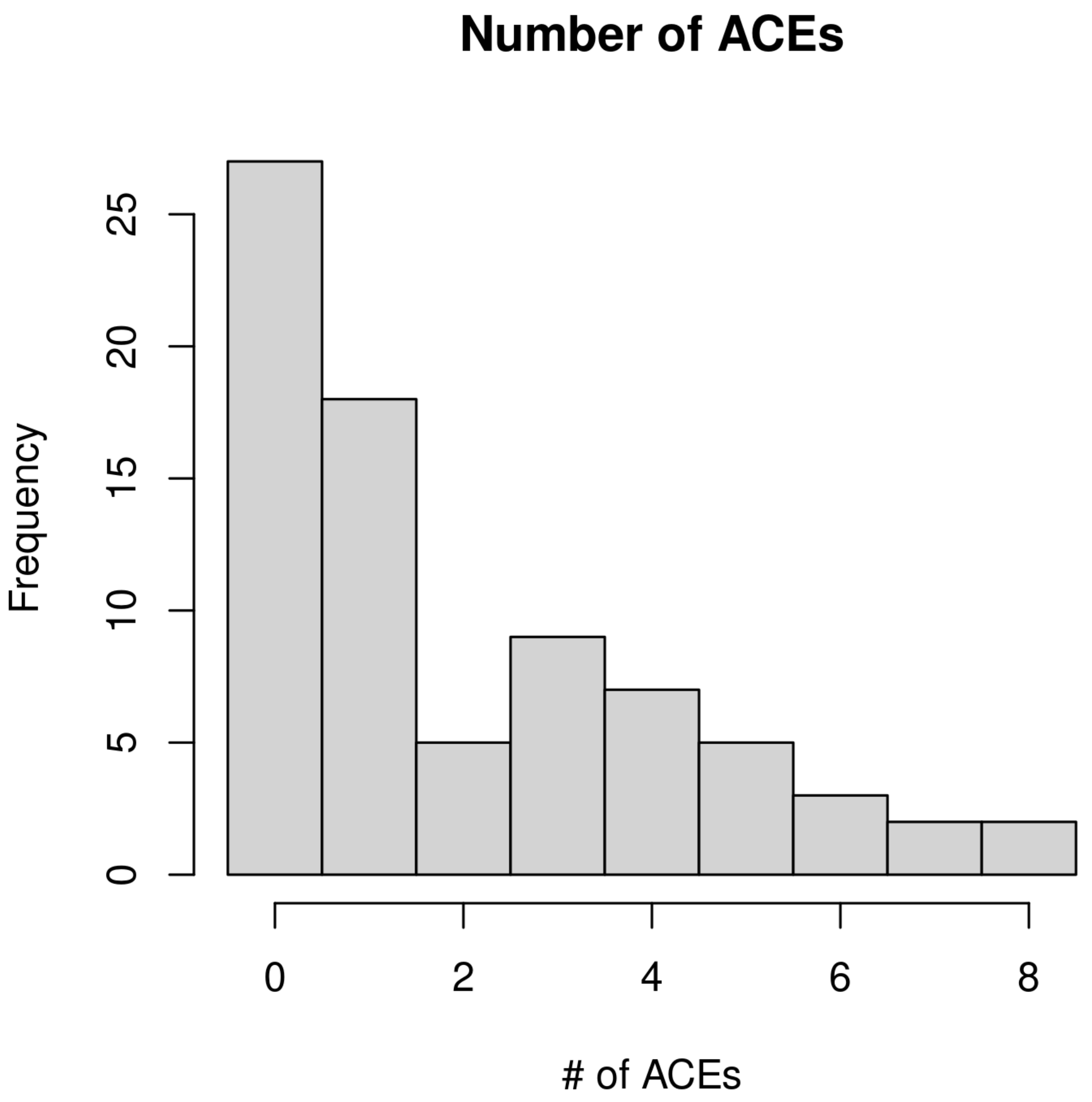

Supplemental Table 2: Models assessing the effect of any prior ACE abuse on NSI-22 sub scale scores after age/gender adjustment.

NSI-22 Total Score

term	estimate	conf.low	conf.high	p.value
Intercept	28.311	14.930	41.693	0.000
Prior Abuse	10.056	2.464	17.648	0.011
Age at Injury (5 years)	-0.135	-1.422	1.151	0.838
Male	2.955	-5.205	11.114	0.480

Cognitive Score

term	estimate	conf.low	conf.high	p.value
Intercept	5.642	2.396	8.888	0.001
Prior Abuse	2.540	0.698	4.381	0.009
Age at Injury (5 years)	0.106	-0.206	0.419	0.506
Male	0.347	-1.632	2.326	0.732

Vestibular Score

term	estimate	conf.low	conf.high	p.value
Intercept	2.318	-0.029	4.665	0.057
Prior Abuse	1.341	0.010	2.673	0.052
Age at Injury (5 years)	0.075	-0.151	0.301	0.516
Male	0.365	-1.066	1.797	0.618

Somatosensory Score

term	estimate	conf.low	conf.high	p.value
Intercept	7.526	2.726	12.326	0.003
Prior Abuse	3.501	0.777	6.224	0.014
Age at Injury (5 years)	-0.119	-0.580	0.342	0.615
Male	0.995	-1.932	3.921	0.507

Affective Score

term	estimate	conf.low	conf.high	p.value
Intercept	12.561	7.340	17.783	0.000
Prior Abuse	2.943	-0.019	5.905	0.055
Age at Injury (5 years)	-0.169	-0.671	0.333	0.512
Male	1.278	-1.906	4.462	0.434

Supplemental Table 3: Models assessing the effect of number of ACEs on NSI-22 scores (admission and subscale scores) after adjusting for age and gender.

NSI-22 Admission

term	estimate	conf.low	conf.high	p.value
Intercept	28.572	16.293	40.852	0.000
# ACE Present	2.847	1.278	4.416	0.001
Age at Injury (5 years)	-0.037	-1.282	1.207	0.954
Male	2.144	-5.637	9.925	0.591

Cognitive Score

term	estimate	conf.low	conf.high	p.value
Intercept	5.859	2.849	8.869	0.000
# ACE Present	0.664	0.279	1.048	0.001
Age at Injury (5 years)	0.127	-0.178	0.432	0.417
Male	0.122	-1.785	2.029	0.900

Vestibular Score

term	estimate	conf.low	conf.high	p.value
Intercept	2.187	0.037	4.338	0.050
# ACE Present	0.440	0.165	0.715	0.002
Age at Injury (5 years)	0.093	-0.125	0.311	0.407
Male	0.279	-1.083	1.642	0.689

Somatic/Sensory Score

term	estimate	conf.low	conf.high	p.value
Intercept	7.996	3.491	12.501	0.001
# ACE Present	0.853	0.277	1.428	0.005
Age at Injury (5 years)	-0.095	-0.552	0.361	0.684
Male	0.662	-2.193	3.516	0.651

Affective Score

term	estimate	conf.low	conf.high	p.value
Intercept	12.378	7.565	17.191	0.000
# ACE Present	0.928	0.313	1.543	0.004
Age at Injury (5 years)	-0.133	-0.621	0.355	0.595
Male	1.075	-1.975	4.125	0.492

Supplemental Table 4: Mediation analysis used to assess potential effect of not including term as an adjustment in model. Only mediator variables that were shown to have significant differences between no prior ACE abuse and prior ACE abuse patients were included in analysis. Estimates in the table below were calculated by taking the difference of prior abuse estimate from model including age/gender adjustment to a model using prior abuse, age, gender, and the specific mediator variable. Original prior abuse estimate comes from model with any prior ACE Abuse, age, and gender.

Original Prior Abuse Estimate

estimate	conf.low	conf.high
10.06	2.46	17.65

Mediation Analysis

Term	Change in Abuse Estimate	Mediator Estimate	Mediator P-Value	Prior Abuse Estimate
History of PTSD	0.83	3.65 (-6.57, 13.87)	0.49	9.23 (1.26, 17.19)
History of Mood Disorder	-0.85	-2.12 (-13.17, 8.94)	0.71	10.91 (2.07, 19.75)
History of Substance Abuse	-1.29	-3.8 (-12.35, 4.74)	0.39	11.34 (3.21, 19.48)
History of Sleep Disorder	-0.40	-1.13 (-8.96, 6.69)	0.78	10.45 (2.34, 18.57)
